# The Impact of Gut Microbiota on Radiation-Induced Enteritis

**DOI:** 10.3389/fcimb.2021.586392

**Published:** 2021-07-29

**Authors:** Yongping Jian, Dan Zhang, Mingdi Liu, Yishu Wang, Zhi-Xiang Xu

**Affiliations:** ^1^Key Laboratory of Pathobiology, Ministry of Education, Norman Bethune College of Medicine, Jilin University, Changchun, China; ^2^School of Life Sciences, Henan University, Kaifeng, China

**Keywords:** microbiota, radiation enteritis, intestinal epithelial barrier, inflammatory cytokines, probiotics

## Abstract

Radiotherapy is an important treatment for abdominal tumors. A critical side effect for this therapy is enteritis. In this review, we aim to summarize recent findings in radiation enteritis, in particular the role of gut microbiota dysbiosis in the development and therapy of the disease. Gut microbiota dysbiosis plays an important role in the occurrence of various diseases, such as radiation enteritis. Abdominal radiation results in changes in the composition of microbiota and reduces its diversity, which is mainly reflected in the decrease of *Lactobacillus* spp. and *Bifidobacterium* spp. and increase of *Escherichia coli* and *Staphylococcus* spp. Gut microbiota dysbiosis aggravates radiation enteritis, weakens intestinal epithelial barrier function, and promotes inflammatory factor expression. Pathogenic *Escherichia coli* induce the rearrangement and redistribution of claudin-1, occludin, and ZO-1 in tight junctions, a critical component in intestinal epithelial barrier. In view of the role that microbiome plays in radiation enteritis, we believe that intestinal flora could be a potential biomarker for the disease. Correction of microbiome by application of probiotics, fecal microbiota transplantation (FMT), and antibiotics could be an effective method for the prevention and treatment of radiation-induced enteritis.

## Highlights

In this article, we reviewed the alteration of intestinal flora and its functional impact on radiation enteritis. Specifically, we summarized changes in the composition and diversity of microbiota in radiation-induced enteritis. We further reviewed the mechanisms by which gut microbiota dysbiosis promotes the development of radiation enteritis. In addition, we summarized recent findings in the treatment of radiation enteritis by correction of microbiome with probiotics and FMT. Our goal is to consolidate the current literature to better delineate the relationship between microbiota and radiation enteritis.

## Introduction

Emil Grubbe’s treatment of breast cancer with radiotherapy back in 1896 (Hodges, 1964), pioneered modern cancer radiation therapy. As the number of patients receiving radiation increases steadily, the incidence of radiotherapy-associated complications has risen in parallel (Theis et al., 2010; Li et al., 2020b). While radiotherapy improves the survival of cancer patients (Hale, 2020), the oxidative stress caused by radiotherapy can produce reactive oxide species (ROS), which result in broad DNA damage to normal tissues, e.g., the gastrointestinal mucosa (Kumagai et al., 2018). It was reported that 6% to 78% of the long-term post-radiation survivors suffer from complications, leading to a compromise in patient’s quality of life (Andreyev, 2005). The most common side effect of this therapy is intestinal mucositis caused by injuries of normal intestinal epithelial cells. Intestinal mucosal atrophy and ulcer caused by radiotherapy hinder the renewal of basal epithelial cells (radiation-induced enteritis) (Sonis et al., 2004; Hauer-Jensen et al., 2014). The incidence of radiation-induced enteritis is largely dependent on radiation treatment scheme (Shaw and Taylor, 2012; Siegel et al., 2012). Patients with acute radiotherapy enteritis manifest symptoms, such as diarrhea, abdominal pain, constipation, hematochezia and loss of weight. Severe radiation-induced enteritis can lead to life-threatening systemic infection over a course of 3 months (Lalla et al., 2014; Marin et al., 2014; Lian et al., 2017). Therefore, prevention of adverse side effects associated with radiotherapy has become an urgent priority (Cui et al., 2019). It was reported that 90% of patients receiving abdominal radiotherapy develop gastrointestinal symptoms in a few weeks after the treatment (Andreyev, 2005). The intestinal microecology is disordered, and the infection of *Clostridium difficile* increases accordingly (Hautmann et al., 2011). In view of the key role of intestinal microecology in diseases, understanding the relationship between radiation enteritis and intestinal microecology and modulating the intestinal microecology to alleviate radiation enteritis may demonstrate a therapeutic benefit.

## Microbiome Functions in Human Body

Intestinal mucosal barriers include those induced by mechanical, chemical, immunological and biological factors (Baumgart and Dignass, 2002). The biological factors refer to microbiome parasitizing in gastrointestinal tract of healthy people. The size of population for microbiome in an average adult intestine is around 100 trillion (Xu and Gordon, 2003). In healthy individuals, the intestinal flora maintains a stable and mutualism relationship with human host, and involves in almost all physiological aspects in nutrition, immune, and digestion. Gut microbiota is beneficial to host homeostasis and immune system development, regulating innate and adaptive immune response (Sommer and Backhed, 2013). The physical condition of host depends on the interaction between the microbiome and the immune system (Barroso-Batista et al., 2015). In addition, gut microbiota also regulates host metabolism. Dysbiosis is related to the occurrence of multiple metabolic diseases, such as type 2 diabetes (Amar et al., 2011) and obesity (Sonnenburg and Backhed, 2016), indicating that a stable gut microbiome is essential to human health.

**Figure 1:**
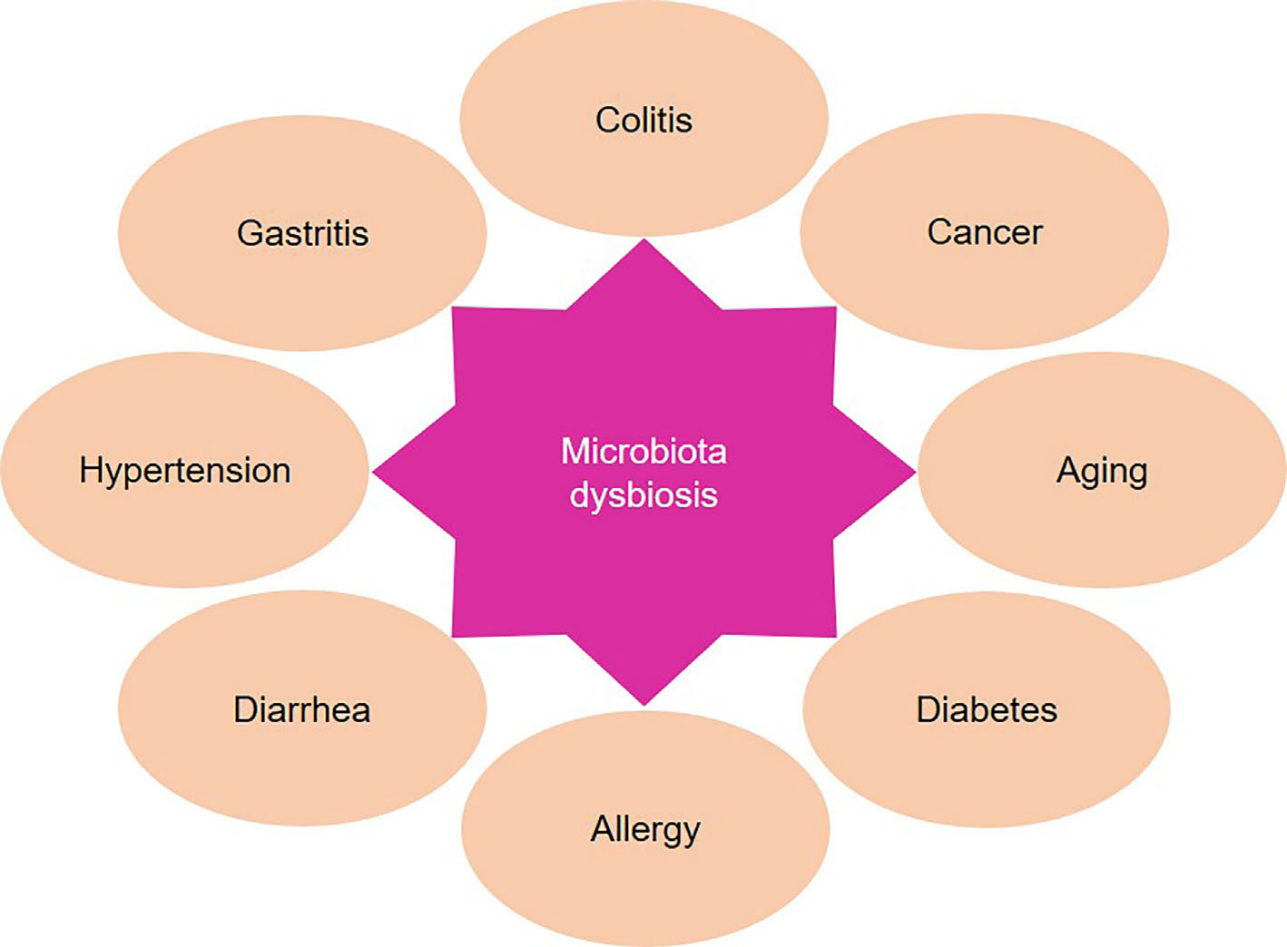
Microbiota dysbiosis is involved in the development of multiple diseases. The intestinal flora affects immune, metabolism and other functions of the host. Microbiota dysbiosis leads to a series of diseases, such as diabetes and colitis.

Gut microbiome dysbiosis is also closely related to the occurrence and metastasis of dietary-associated cancers (Schulz et al., 2014; Feng et al., 2015). Intestinal flora may be used as biomarkers for the diagnosis of cancer. For example, the development of gastric cancer is associated with dysbacteriosis. The microbial dysbiosis index improves the sensitivity and specificity in detecting gastric cancer, indicating that changes in the overall flora rather than changes in individual flora lead to gastric cancer (Ferreira et al., 2018). In addition, *Fusobacterium* and related bacteria may also promote the growth and metastasis of colorectal cancer. Microbiota co-exists in primary and matched metastatic tumors, and is continuously associated with distant metastasis of primary human colorectal cancer (Bullman et al., 2017). Microbiome is also strongly linked to cancer treatment. Reports showed that the composition of gut microbiota can impact anti-cancer treatment by improving antitumor immunity. Targeting gut microbiota strengthens the efficacy of drugs and reduces adverse reactions (Gorjifard and Goldszmid, 2015; Roy and Trinchieri, 2017) (**Figure 1**).

Radiotherapy remains to be essential for most of cancers. The imbalance in the diversity of microbiome is associated with the occurrence and treatment failure of a variety of diseases, including radiation enteritis (Sokol and Adolph, 2018). It is necessary to clarify the interaction between intestinal flora and radiation-induced enteritis to provide optimal treatment for patients.

## Pathogenesis of Radiation-Induced Enteritis: Emerging Roles of Gut Microbiota

The symptoms of radiation-associated enteritis, such as diarrhea, are correlated with the onset time and duration of radiotherapy (Hogan and Kellison, 2002; Keefe, 2007). It has been reported that radiotherapy-induced enteritis is a common reason for reduced dosage of irradiation in radiotherapy. Such reduction can severely compromise body functions and increase mortality among cancer patients (van Vliet et al., 2010; Tarricone et al., 2016). High doses of radiotherapy lead to intestinal villi atrophy, intestinal epithelial damage, increased apoptosis, and spike in inflammation. Moreover, radiotherapy can also result in the dysfunction of intestinal epithelial barrier, leading to increased intestinal permeability, diarrhea, and disruption of water and electrolyte processing. This may ultimately result in hypovolemic shock, hence endangering life (Barnett et al., 2006; Shen et al., 2018). Radiation enteritis includes five phases, (i) the initial phase with the formation of ROS that result in DNA damage, (ii) the primary damage response phase with inflammation and apoptosis, (iii) the signal amplification phase, in which more inflammation and apoptosis occurs, (iv) the phase of ulcer formation, with discontinuity of the epithelial barrier that promotes bacterial translocation, and (v) the healing phase, with cell proliferation once radiotherapy has ceased (Sonis, 2004). Although recent studies have begun to reveal the pathogenesis of radiation enteritis, our current knowledge is far from being able to provide an effective prevention or treatment for the disease.

There is evidence that the pathophysiology of intestinal injury caused by radiation is related to the dysbiosis of gut microbiota. To demonstrate the influence of gut microbiota on intestinal inflammation, García-Lafuente et al. evaluated the effect of colonic microbiota on 2,4,6-trinitrobenzene sulfonic acid (TNBS)-induced intestinal inflammation in rats. Animals whose colon segments were excluded from fecal transport were recolonized with pre-selected bacteria to test the effects of different species on intestinal inflammation and damage. Rats with excluded colon and sterile culture of intracavitary washings showed slight inflammation and low mucosal damage in response to TNBS. Rats colonized with anaerobic bacteria showed significantly higher release of eicosanoids than rats colonized with aerobic bacteria only. In addition, mucosal lesions were mainly observed in rats with anaerobic bacteria, which suggest that colonic anaerobic bacteria may play a role in intestinal inflammation (García-Lafuente et al., 1997). Therefore, it is necessary to clarify the relationship between radiation intestinal injury and intestinal flora. We will give an overview of the characteristics of alteration of intestinal microbiota in radiation enteritis and its possible mechanisms in radiotherapy-induced intestinal injury.

Although limitations exist in studies with animals, the majority of the intestinal flora in mice is unique, with high similarities to those in humans (e.g., phylum level of both mice and humans are mainly dominated by *Firmicutes* and *Bacteroidetes* (Ley et al., 2005). Animal models, if used within ethical bounds, are crucial in identifying the relationship between radiotherapy enteritis and gut microbiota (Jones et al., 2020). We will not only summarize the changes of microbiome in clinical patients with radiation enteritis, but will also discuss the relationship between radiation enteritis and intestinal flora in animal models.

## Radiation Leads to Alterations in the Composition and Diversity of Gut Microbiota

The development and application of 16S rRNA sequencing greatly promote the study of gut microbiota. 16S rRNA sequencing is a cost-effective method to study the microbiota diversity and composition in a large number of samples. However, this method is limited to the detection in flora composition. In order to obtain a comprehensive understanding of the microbiota, combined applications of 16S rRNA sequencing with metabolomics, transcriptomics, shotgun metagenomics, and other methods are recommended (Goudarzi et al., 2016).

Cui et al. determined the relationship between microbiome and the radiation sensitivity in the radiation enteritis through the 16S rRNA sequencing, and found that 6.5 Gy gamma ray with whole body radiation changed community composition of intestinal microbiota in C57BL/6 mice. The authors further confirmed that alterations in intestinal flora affect post-irradiation survival of mice mainly due to microbiome changes, which deregulate lncRNA expression of the host, hence sensitizing the mice to radiation (Cui et al., 2017). In addition, Johnson et al. found that approximately two hours after radiation, *aerobic bacteria* and *Lactobacillus* counts in the intestine become markedly reduced (Johnson et al., 2004). Zhao et al. obtained a similar result, proving that abdominal radiation disrupted the intestinal flora balance and significantly reduced gut microbiota diversity in mice (Zhao et al., 2020). Together, current studies demonstrate that in animal models, abdominal radiation induces gut microbiota dysbiosis and reduces the survival of radioactive mice.

Clinically, the incidence of grade 2 or even worse diarrhea was as high as 17% in patients undergoing radiotherapy before surgery (Bosset et al., 2004). Excessive growth of gram-negative bacilli is detected in radiation-enteritis-induced diarrhea. Comparison of microbiota immediately before, immediately after, and two weeks after radiotherapy indicated that patients with diarrhea showed an increase in *Actinobacteria* phylum and a reduction in *Clostridium*. Impairment of intestinal motility after radiation is the main pathogenic factor for colonization of gram-negative bacilli in gastrointestinal tract. During bacterial overgrowth, absence of intestinal migrating motor complex (MMC) correlates with the existence of mass colonization of gram-negative bacilli (Bosset et al., 2004). Thus, abnormal intestinal motility and mass colonization of gram-negative bacilli are important factors for the development of severe late-stage radiation-induced enteropathy. In addition, the susceptibility and protection to diarrhea and other bacterial changes after radiotherapy may also be related to different initial microbiota colonization (Husebye et al., 1995; Manichanh et al., 2008).

Manichanh et al. analyzed the intestinal bacteria of patients receiving radiotherapy (Manichanh et al., 2008; Stringer et al., 2013). In radiation enteritis patients without diarrhea, *Actinobacteria* phylum was not detected, whereas in patients with diarrhea, *Bacilli class* showed a higher abundance. Radiation therapy reestablishes the intestinal flora, and acute diarrhea induced by radiation enteritis is associated with the reconstitution of colonized intestinal flora (Manichanh et al., 2008; Stringer et al., 2013). Differences in the level of gut microbiota diversity reveal an obvious variance in microbiota composition and structure between cancer patients and healthy individuals. Stool samples were collected from 18 patients with cervical cancer during radiotherapy. Wang et al. used the Illumina HiSeq platform to characterize the microbiota based on 16S rRNA sequencing. Microbiota dysbiosis was observed in radiation enteritis patients with a significant decrease in α diversity and an increase in β diversity. The abundance of *Proteobacteria*, *Gamma proteobacteria* and *Coprococcus* was increased, while the level of *Bacteroide*s was reduced (Wang et al., 2019).

Manichanh et al. demonstrated that there are different intestinal floras in abdominal cancer patients and in healthy individuals, because shifts in intestinal health status (such as chronic inflammation or abnormal function of epithelial cells) may directly affect microbiome (Manichanh et al., 2008). There is no direct causal relationship between gynecological cancer and intestinal flora, but Nam et al. found that there were significant differences in gut microbiota between gynecological cancer patients and healthy individuals. As compared with healthy individuals, gynecologic cancer patients receiving radiation therapy mainly possess a relative abundance of the phylum. *Actinobacteria* were 30 times higher than those in healthy individuals whereas *Bacteroidetes* and *Fusobacteria* were lower than those in healthy people. At the family level, the relative abundance of *Eubacteriaceae* was obviously higher, and *Prevotellaceae*, *Oscillospiraceae* and *Fusobacteriaceae* were lower than those in healthy group (Nam et al., 2013). Therefore, even if the location of disease development is far from or not related to the intestinal tract, the alteration in health status of the host will also affect the overall homeostasis of intestinal flora. Gut microbiota in different types of cancer patients may also be different as compared with healthy individuals.

Together, current studies suggest that gut microbiota in radiation-associated enteritis patients show marked changes in terms of composition and diversity. In radiation enteritis, the abundance of most bacteria belonging to the phylum *Actinobacteria* and *Proteobacteria* increases, and most of these bacteria are conditional pathogens, such as *Escherichia coli*. In contrast, microorganisms from *Firmicutes* and *Bacteroides* are reduced. Most of these bacteria are probiotics, such as *Lactobacillus*. When the abundance of probiotics decreases, conditional pathogens will reproduce and occupy the niche, which, in return, further inhibit the growth of probiotics and promote the release of endotoxin, and hence enhance intestinal inflammation. A vicious cycle may thus exacerbate radiation enteritis (**Table 1**).

**Table 1:** Radiotherapy affects the composition and diversity of intestinal microbiota.

Samples	Clinical setting	Techniques used	Disease	Alterations in the gut microbiota composition	References
Feces of male C57BL/6J mice	A single dose of 6.5 Gy gamma ray at a rate of 1.0 Gy/min	16S rRNA sequencing	–	*Bacteroides↓*	Cui et al., 2017
Irradiated ileals of male C57BL/6J mice	Ileal *in vitro* absorbed dose of 19 Gy and dose rate was 3.2 Gy/minute	37°C incubate	–	*Aerobic bacteria↓*	Johnson et al., 2004
				*Lactobacillus↓*	
Feces of male C57BL/6J mice	High-dose abdominal precision radiation with a single dose of 10 Gy	16S rRNA sequencing	–	*Proteobacteria↑*	Zhao et al., 2020
				*Bacteroidete↓*	
				*Firmicutes↓*	
				*Actinobacteria↓*	
				*Verrucomicrobia↓*	
Culture of gastric and duodenal samples	Abdominal radiotherapy	Glucose gas test and [14C] D-xylose breath test	Gynecologic cancer	*Gram-negative bacilli↑*	Husebye et al., 1995
Feces of patients	Abdominal radiotherapy	16S rRNA sequencing	Abdominal tumor	*Bacilli↑*	Manichanh et al., 2008
				*Actinobacteria↑*	
				*Clostridia↓*	
Feces of patients	Pelvic radiotherapy	16S rRNA sequencing	Cervical cancer	*Proteobacteria↑*	Wang et al., 2019
				*Gammaprote-obacteria↑*	
				*Coprococcus↑*	
				*Bacteroides↓*	
Feces of patients	Pelvic radiotherapy, five times a week during a 5 weeks period	454 pyrosequencing	Gynecologic cancer	*Gram-negative bacilli↑*	Nam et al., 2013
				*Eubacteriaceae↑*	
				*Prevotellaceae↓*	
				*Oscillospiraceae↓*	
				*Fusobacteriaceae↓*	

The meaning of a symbol in the table: ↑, increased; ↓, decreased.

## Mechanisms by Which Dysbiosis of Gut Microbiota Contributes to Radiation-Induced Enteritis

Radiation-induced enteritis refers to the intestinal mucosa damaged by the free radicals derived from ionization. Radiation enteritis is manifested by phenotypes such as impaired intestinal mucosal barrier, increased inflammatory factors, enhanced pathogen invasion and endotoxin release, and decreased immune barrier.

### The Gut Microbiota Dysbiosis Weakens the Function of Intestinal Epithelial Barrier

The intestinal epithelial barrier is composed of mucus layer, epithelial glycoglobulin and epithelial cells, and plays a critical role in the prevention of pathogen invasion (Sánchez de Medina et al., 2014). As an important component of the intestinal epithelial barrier, intercellular junctions, which include tight junction, gap junction, adhesion junction and desmosome, form multiple functional complexes (Anderson and Van Itallie, 2009; Groschwitz and Hogan, 2009). Tight junction, as the most important intercellular junction, determines the intestinal epithelial permeability and maintains physiological functions of the intestinal barrier (Zihni et al., 2016). Zonula occludens-1 (ZO-1), occludin, and claudins are three most important tight junction proteins, which play a vital role in maintaining cell polarity and intestinal epithelial barrier (Bazzoni et al., 2000). It was found that tight junction proteins are essential in repairing intestinal epithelial damages. Accumulating evidence shows that intestinal microbiome involves in the intestinal epithelial cell signaling and affects the intestinal barrier function (Ulluwishewa et al., 2011). Gut microbiota regulates intestinal barrier function by maintaining tight junction protein expression and distributions, which are important in intestinal barrier integrity (Ulluwishewa et al., 2011).

The intestinal epithelial mucus is a protective layer, which is regulated by bacteria such as *Lactobacilli*, *Bifidobacteria*, and *Streptococci* that play a positive role in strengthening the intestinal mucosal barrier. In the rat model, microbiota markedly promotes the expression of MUC2 gene and secretion of colonic MUC2 (Caballero-Franco et al., 2007). *Akkermansia muciniphila* is a type of gram-negative intestinal symbiotic bacteria that also promotes intestinal barrier function by enhancing mucous generation. Short-chain fatty acids (SCFAs) produced by *Akkermansia muciniphila* enter the intestinal epithelial cells through G protein-coupled receptor (GPCR) 41/43 to increase the expression of tight junction protein claudin-3 and occludin (Le Poul et al., 2003; Maslowski et al., 2009; Grander et al., 2018). Administration of *Lactobacillus* to healthy subjects significantly increases the scaffold protein ZO-1 and occludin in the vicinity of the tight junction structure, forming a cell side seal between the epithelial cells (Karczewski et al., 2010).

In radiation-associated enteritis patients, probiotics can also maintain micro-ecological stability and protect the intestinal barrier function by interrupting the pathogen’s infection or inhibiting the growth of pathogens. After intestinal pathogenic *Escherichia coli* infection, changes in host cell cytoskeleton reduce the absorbing surface of intestinal epithelial cells, leading to pathogenic *Escherichia coli* infection-associated persistent diarrhea. However, *Lactobacillus plantarum* prevents pathogenic *Escherichia coli*-induced rearrangement and redistribution of claudin-1, occludin, ZO-1 and JAM-1 (Qin et al., 2009). *Lactobacillus* competes with pathogens to bind to receptors on the surface of intestinal epithelial cells, inhibits pathogen growth, and reduces the vitality and toxicity of pathogenic *Escherichia coli* (Sherman et al., 2005). In addition, certain harmful bacteria in *Enterobacteriaceae* can form a biofilm on the surface of the epithelium, altering and destroying the mucus layer (Hansson and Johansson, 2010). The increase of *Citrobacter rodentium* digests mucins with glycosidase, and participates in the degradation of mucus barrier (Bergstrom et al., 2010). Thus, the colonization of probiotics decreases, and the pathogenic bacteria multiply without competitors. The imbalance of intestinal flora homeostasis will weaken the intestinal epithelial barrier integrity, resulting in increased intestinal permeability.

### Microbiome Dysbiosis Contributes to the Expression of Inflammatory Cytokines

Grander et al. found that recombination with *Akkermansia muciniphila* reduces expression of IL-1β and TNF-α and decrease the infiltration of MPO+ neutrophils in mice (Grander et al., 2018). Gut probiotics inhibit inflammation *via* suppressing the activation of NF-κB and TNF-α. This signaling pathway plays an important role in intestinal microbiota balance, which helps to regulate intestinal homeostasis, maintains intestinal barrier function and promotes repair and regeneration of tissues damaged (Stringer, 2013). *Ruminococcus*, *Coprococcus*, *Dorea*, *Lachnospira*, *Roseburia*, *Bifidobacterium* and *Clostridium* can alleviate inflammation by inhibiting the TLR-NF-κB pathway (Lakhdari et al., 2011). *Bifidobacterium* inhibits intestinal epithelial cell inflammation by attenuating the inflammatory response induced by TNF-α and lipopolysaccharide (LPS) (Riedel et al., 2006). In addition, increased *Citrobacter* can activate NF-κB (Wang et al., 2006), and also involve in the degradation of the mucus barrier, thus increasing the inflammatory response. NF-κB and TNF-α are able to increase the production of myosin light-chain kinase, resulting in the disassembly of tight junction proteins. *Biffidobactrium* diminishes the formation of intestinal endotoxins and increases the production of tight junction proteins, thus decreasing intestinal permeability and bacterial translocation (Ewaschuk et al., 2008). Microbiome dysbiosis induced by radiation enteritis leads to severe side effects in immunodeficiency cancer patients because intestinal flora plays a vital role in innate and adaptive immune responses of hosts (Maynard et al., 2012). Interestingly, less apoptosis was observed in endothelial cells and lymphocytes in small intestine of germ free (GF) mice received a lethal dose of irradiation, demonstrating that GF mice may be resistance to lethal radioactive enteritis (Crawford and Gordon, 2005). Together, these studies suggest that microbiota dysbiosis may be detrimental to the maintenance of effective intestinal barrier function after radiation.

**Figure 2:**
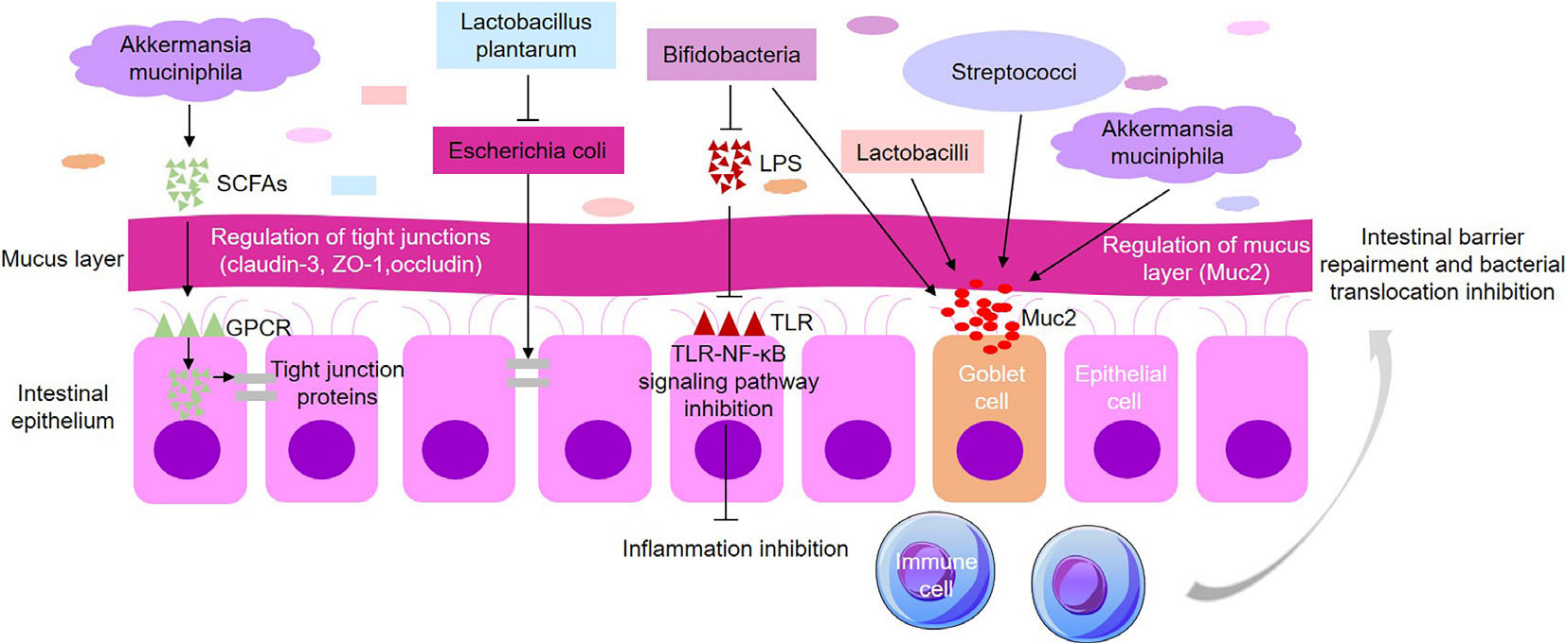
Gut microbiota affects the function of intestinal epithelial barrier. Different types of microbiota act on various targets associated with intestinal epithelial barrier. *Biffidobactrium*, *Lactobacilli*, *Streptococci* and *Akkermansia muciniphila* stimulate the secretion of MUC2. SCFAs produced by *Akkermansia muciniphila* enter into the intestinal epithelial cells through GPCR to increase the expression of tight junction protein claudin-3 and occludin. *Lactobacillus plantarum* inhibits pathogenic *Escherichia coli* growth and increases the expression of ZO-1 and occludin. *Biffidobactrium* diminishes the formation of LPS and inhibits the TLR-NF-κB signaling to alleviate inflammation, hence decreasing intestinal permeability and bacterial translocation.

The mechanisms by which changes in the gut microbiota affect intestinal injury in radiation are expected to be extremely complicated. The dynamic changes of the microbial community interact with various components within the intestine rather than a single compound in the system. Current evidence supports and illustrates that intestinal microbiome plays a critical role in radiation-associated enteritis *via* up-regulating key regulatory factors. Gut microbiota and the body form a symbiotic and stable relationship in the protection of the intestinal epithelium, reduction of inflammatory responses, and maintenance of normal immune tolerance in order to sustain host’s immune homeostasis, growth, and development (Sender et al., 2016). If there is an imbalance in intestinal flora, the relative abundance and diversity of the probiotics will be severely compromised, leading to limited intestinal barrier function, harmful bacteria overgrowth, accumulation of endotoxin in the blood, increased inflammatory factors, and eventually aggravating radiotherapy-induced enteritis. Thus, a stabilized intestinal microbiota is crucial for radiation enteritis (**Figure 2**).

## The Role of Intestinal Flora in the Treatment of Radiotherapy-Induced Enteritis

### Probiotics Regulate the Intestinal Microbiota to Improve Radiation Enteritis

Recent studies have shown that probiotics are beneficial in strengthening the number and activity of host T cells, directly affecting the immune response mediated by T cells and improving immune functions (Redman et al., 2014). Furthermore, probiotics promote the diversity of gut microbiota, and increase the species of intestinal flora, ensuring the integrity of intestinal mucosal barrier so as to avoid bacterial translocation (Reiff and Kelly, 2010). The probiotic VSL# 3 is a mixture of 8 probiotics, which has been used broadly for many years with high safety. Clinically, it was proved that VSL#3 alleviates intestinal inflammation and enhances intestinal barrier functions by improving intestinal flora balance (Mimura et al., 2004; Sartor, 2006; Tankou et al., 2018). Treatment with VSL# 3 probiotics increases the diversity of bacterial community in patients, reduces fungal variety, and elevates the abundance of *Lactobacillus* and *Bifidobacterium*, and hence reduces the incidence and severity of radiotherapy enteritis-associated diarrhea (Kuhbacher et al., 2006; Chitapanarux et al., 2010).

A randomized double-blind controlled trial showed that a standard dose of *Lactobacillus acidophilus LAC-361* and *Bifidobacterium longum BB-536* decreased level 2, 3, and 4 diarrhea caused by radiation enteritis in patients with surgery. Demers et al. analyzed a total of 229 patients and found that for patients who had undergone surgery, the incidence of diarrhea was reduced after probiotic treatment. For patients who did not undergo surgery, there was no statistical difference (Demers et al., 2014). In fact, for patients without surgery, patients treated with standard doses of probiotics had fewer moderate to severe diarrhea than the placebo group at the end of radiotherapy. For patients with surgery before radiotherapy, administration of probiotics tends to reduce all degrees of diarrhea, especially the most severe grade 4 diarrhea. These results may be attributable to the individualized nutritional interventions during the treatment, and these interventions were adjusted as the treatment progressed. It is difficult to compare these results with other published studies due to different treatment options. In addition, compared to probiotic treatment group, the placebo group showed a significant destruction of gut microbiota after radiotherapy enteritis (e.g., through an increase in *Enterobacteriaceae* (Osterlund et al., 2007). The intragastric administration of *Lactobacillus rhamnosus* increased the crypts survival in radiation-induced enteritis by approximately two-fold and reduced epithelial cell apoptosis at the crypt basement, which depends on intact Toll-like receptor 2 (TLR2) and MyD88-dependent signals that involve in the relocation and expression of cyclooxygenase 2 (COX-2) in mesenchymal stem cells of crypt region (Ciorba et al., 2012; Uribe et al., 2018; Riehl et al., 2019). *Bifidobacterium infantis* produces indole-3-lactic acid (ILA) to protect intestinal epithelial cells in culture *via* activation of the aryl hydrogen receptor (AhR) and nuclear factor erythroid 2-related factor 2 (Nrf2) (Ehrlich et al., 2020). Thus, prebiotics and synbiotics can also promote the growth and reproduction of probiotics and hence reduce diarrhea in radiotherapy patients (Garcia-Peris et al., 2016).

Probiotics-mediated homeostasis of intestinal microbiota may also prevent other symptoms from radiation enteritis, but more studies are needed to clarify the observation, which is the key to maintaining the biological strategies of healthy intestine during radiotherapy of cancer (Bentzen, 2006; Fuccio and Guido, 2013; Florez et al., 2016).

### Fecal Microbiota Transplantation Maintains the Balance of Gut Microbiota to Improve Radiotherapy-Induced Enteritis

FMT is a treatment method to reestablish the gut microbiota of patients by separating and transplanting the intestinal flora of healthy persons into patient’s intestine tract (Xu et al., 2016). In 1958, Eiseman et al. applied FMT to treat Pseudomembranous enteritis caused by the treatment of antibiotics, leading to an improvement of patient’s condition (Eiseman et al., 1958). With the analysis of the 16S rRNA sequencing, Cui et al. confirmed that FMT treatment increases the relative abundance of the intestinal microbiota, such as escalation (or stabilization) of *Bacteroidetes* (or *Lactobacillus*), augment of *Prevotella* at the genus level. In addition, FMT-improved gut microbiota can reduce the intestinal leakage and enhance the intestinal functions and epithelial integrity in radiotherapy-induced enteritis (Cui et al., 2017). Ding et al. proved that the intestinal flora diversity of all patients underwent radiotherapy increased after FMT treatment. Radiation-induced intestinal edema was strikingly alleviated after eight weeks of FMT, and the beneficial bacteria such as *Alistipes*, *Phascolarctobacterium*, *Streptococcus* and *Bacteroides* expanded, whereas the abundance of *Faecalibacterium* decreased (Ding et al., 2020). FMT of gut microbes from healthy donors may reduce the toxicity caused by radiotherapy (Cui et al., 2017). In addition, FMT could elevate the level of microbial-derived indole 3-propionic acid (IPA) in the feces of irradiated mice, so that the mice had a lower level of systemic inflammation, a reduced hematopoietic organ injury and catabatic myelosuppression, and improved gastrointestinal tract functions and epithelial integrity (Xiao et al., 2020). Li et al. proved that FMT increased the level of SCFAs in feces and valeric acid (VA) produced by microbiota exerted the most important radioprotective effect by increasing the expression of keratin 1 (KRT1). VA supplementation increased the survival of irradiated mice, protected their hematopoietic organs, and alleviated gastrointestinal injury of irradiated mice (Li et al., 2020a).

Fresh stool used for bacterial transplantation can effectively treat *Clostridium difficile* infection. For patients with severe *Clostridium difficile* infection, multiple stool transplantation may effectively relieve diarrhea (Hui et al., 2019). FMT capsules were also used to treat pediatric diarrhea and other diseases. It significantly improves both overall and gastrointestinal health after FMT (Youngster et al., 2014). FMT also has an obvious effect on the treatment of pediatric inflammatory bowel disease (IBD). Patients prior to FMT treatment exhibited reduced biodiversity and significantly differed in gut microbiota composition characterized by an increase in *Enterobacteriaceae*, *Enterococcus*, *Haemophilus*, and *Fusobacterium* compared to donors, and an augment in species diversity at 30 days post-FMT treatment (Goyal et al., 2018). Paramsothy et al. demonstrated that microbiota diversity increased with the persistence of FMT, which could alleviate the clinical symptoms of ulcerative colitis, and was mainly associated with the improvement of microbial diversity (Paramsothy et al., 2017). Ishikawa et al. also confirmed that FMT is a potential treatment for restoring normal intestinal flora in patients with ulcerative colitis (Ishikawa et al., 2017). Selection of healthy donors for FMT may avoid potential adverse effects transmitted from donor’s feces in the therapy of radiation-induced enteritis. Standardization of this technology and humanization of FMT are two key factors in the usage of the therapy to meet the needs of patients (Ren et al., 2016).

### Antibiotics Reconstruct the Intestinal Microbiota to Alleviate Radiation-Induced Enteritis

Abdominal radiation disrupts the intestinal microbial balance. It reduces microbiota diversity, and increases the relative abundance of pathogenic bacteria, such as *Proteobacteria* in mice. Antibiotic cocktail (ABX) and metronidazole pretreatment are beneficial to the reconstruction of gut microbes in irradiated mice. It has been reported that Abx pretreatment effectively reduces the level of LPS in the ileum and inhibits the TLR4/MyD88/NF-κB signaling, thereby reducing intestinal inflammation. In addition, Abx pretreatment regulates macrophage polarization in the ileum and downregulates the expression of TGF-β1 and phosphorylated Smad-3 and α-SMA thereby preventing intestinal fibrosis and ultimately improving the survival of mice with radiation-induced intestinal injury (Cui et al., 2017; Zhao et al., 2020). These results indicate that antibiotic pretreatment can effectively alleviate gut microbial disorders and intestinal injury caused by abdominal radiation. In general, these findings have increased our understanding of the radiation enteritis pathogenesis.

## Conclusions and Prospects

The intestinal flora is affected by multiple factors, as well as original diseases, such as cancers. Although the location of a disease is far from or not related to the intestinal tract, alterations in health status of the host may also affect the overall homeostasis of intestinal flora. Thus, gut microbiota in various cancer patients may be different as compared with healthy individuals. Studies with strictly selected healthy people for comparison may help to uncover bacterial genera changed in radiation enteritis. In terms of treatment, with intestinal flora in healthy people as references, those in patients with radiation-induced intestinal injury can be manipulated to a comparable level in healthy individuals.

In the current review, we emphasized the relationship between microbiota and radiation enteritis. Radiation reduces the diversity of intestinal flora and modifies gut microbiota composition. We summarized the types of bacteria, which have been changed in radiation enteritis. Alterations in the composition of the intestinal flora aggravate radiation enteritis, which may form a vicious circle and amplify the changes in microbiota. Radiation enteritis could weaken the intestinal epithelial barrier and increase the expression of inflammatory factors. Mounting evidence shows that intestinal microbiota is the initiator for the pathogenesis of radiation enteritis. Targeted treatment of gut microbiota with probiotics, fecal bacteria transplantation and antibiotics can alleviate radiation enteritis. In addition, intestinal flora could be a potential biomarker for radiation enteritis and may help to establish a personalized radiation treatment plan. Further studies regarding the role of intestinal flora in radiation enteritis are needed in order to set up a more reliable preventive strategy for the disease. In the future, on the basis of 16S rRNA sequencing, metabolomics, transcriptomics, shotgun metagenomics, and other state-of-art methods, functions of gut microbiota in radiation enteritis are expected to be explored more extensively. Reversing the changes in microbiome may eventually prove to be an effective therapeutic strategy for patients with life-threatening radiation enteritis.

## Author Contributions

YJ wrote the manuscript. Z-XX, YW, DZ, and ML contributed to critical revision of the paper. All authors contributed to the article and approved the submitted version.

## Funding

This work was supported by the National Natural Science Foundation of China (Nos. 81573087, 81772924, and 82020108024) and International Cooperation Foundation Grant of Jilin Province (20190701006GH).

## Conflict of Interest

The authors declare that the research was conducted in the absence of any commercial or financial relationships that could be construed as a potential conflict of interest.

## Publisher’s Note

All claims expressed in this article are solely those of the authors and do not necessarily represent those of their affiliated organizations, or those of the publisher, the editors and the reviewers. Any product that may be evaluated in this article, or claim that may be made by its manufacturer, is not guaranteed or endorsed by the publisher.
